# K-Ras stabilization by estrogen via PKCδ is involved in endometrial tumorigenesis

**DOI:** 10.18632/oncotarget.4049

**Published:** 2015-05-08

**Authors:** Kyoung-Hwa Koo, Woo-Jeong Jeong, Yong-Hee Cho, Jong-Chan Park, Do Sik Min, Kang-Yell Choi

**Affiliations:** ^1^ Translational Research Center for Protein Function Control, Yonsei University, Seoul, Korea; ^2^ Department of Biotechnology, College of Life Science and Biotechnology, Yonsei University, Seoul, Korea; ^3^ Department of Molecular Biology, College of Natural Science, Pusan National University, Pusan, Korea

**Keywords:** endometrial cancer, estrogen, tumorigenesis, K-Ras, PKCδ

## Abstract

Estrogens are considered as a major risk factor of endometrial cancer. In this study, we identified a mechanism of tumorigenesis in which K-Ras protein is stabilized via estrogen signaling through the ER-α36 receptor. PKCδ was shown to stabilize K-Ras specifically via estrogen signaling. Estrogens stabilize K-Ras via inhibition of polyubiquitylation-dependent proteasomal degradation. Estrogen-induced cellular transformation was abolished by either K-Ras or PKCδ knockdown. The role of PKCδ in estrogen-induced tumorigenesis was confirmed in a mouse xenograft model by reduction of tumors after treatment with rottlerin, a PKCδ inhibitor. Finally, levels of PKCδ correlated with that of Ras in human endometrial tumor tissues. Stabilization of K-Ras by estrogen signaling involving PKCδ up-regulation provides a potential therapeutic approach for treatment of endometrial cancer.

## INTRODUCTION

Endometrial cancer (EC) is the most common type of cancer of the female reproductive system. EC can be classified into two major types: the estrogens-dependent type I endometrioid adenocarcinoma, which represents 70-80% of EC cases, and the estrogens-independent type II non-endometrioid cancers [1]. Genetic alterations commonly observed in type I EC include *PTEN*, *K-Ras*, *PIK3CA,* and *β-catenin*, whereas *p53* and *HER-2/neu* mutations are prevalent in Type II EC [2]. Because estrogens are a major risk factor for EC, progestin hormone to inhibit estrogen action has been used for treatment of EC [3]. Long-lasting unopposed estrogen exposure leads to endometrial hyperplasia, which increases the chance for development of EC [4]. In fact, EC patients retain higher circulating estrogen levels compared with healthy women [5, 6].

Estrogen signaling is activated by both genomic and non-genomic signaling [7]. Genomic estrogen signaling is activated by the direct actions of nuclear-localized estrogen receptors (ERs: ER-α and ER-β), which act as transcription factors [8]. On the other hand, non-genomic signaling involves activation of estrogen-signaling cascades initiated by ligand binding at the plasma membrane [9, 10]. ER-α36 is a novel variant of ER-α66 that lacks both AF-1 and AF-2 transcriptional activation domains. ER-α36 is localized predominantly on the plasma membrane and mainly activates non-genomic estrogen signaling [11], and estrogen-induced proliferation of EC cells. Membrane-associated protein kinase C δ (PKCδ) was shown to be involved in this process [12].

PKCδ is a serine/threonine kinase of the PKC family that functions in proliferation, survival, and apoptosis of cells [13]. In EC cells, total PKC activity was significantly higher than in normal endometrial tissue [14]. In addition, Ras protein levels were higher in primary endometrial adenomas than in normal endometrial tissue [15], indicating a potential role for Ras up-regulation in the development of human EC.

Recently, we found that Ras protein is stabilized via the activated Wnt/β-catenin signaling and stabilization of oncogenic Ras by aberrant mutational activation of Wnt/β-catenin pathway genes related with colorectal tumorigenesis [16-21]. However, other signaling pathways regulating Ras stability and its involvement with human cancer have not been characterized.

Here, we determined that Ras stability was increased by the natural estrogen 17β-estradiol (E_2_) through ER-α36. The K-Ras stabilization by estrogen was enhanced specifically through stabilization of PKCδ. Furthermore, stabilizations of both K-Ras and PKCδ occurred through inhibition of polyubiquitylation-dependent proteasomal degradation. Estrogen-induced K-Ras stabilization via PKCδ contributes endometrial cellular transformation and endometrial tumor growth. Overall, controlling PKCδ and its binding to K-Ras could be a potential therapy for human EC.

## RESULTS

### Estrogens stabilize Ras via ER-α36

**Figure 1:**
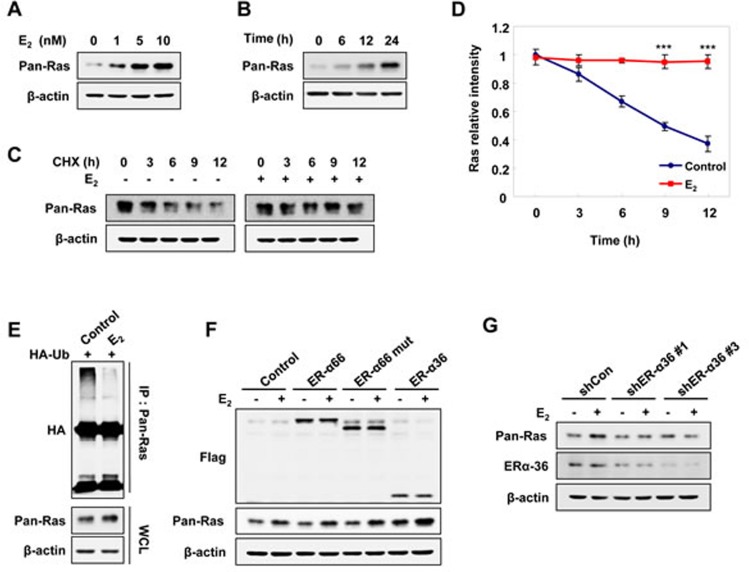
E_2_ induces Ras protein stabilization via ER-α36 in EC cells Ishikawa cells were treated E_2_ in a dose- **A.** and time- **B.** dependent manner. **C.** Ishikawa cells were treated CHX, 50 μg/ml and with/without E_2_ (10 nM) for the indicated time periods. **D.** The graph shows the quantification of the pan-Ras intensities of the blot on **C.** relative to β-actin control. The band intensity of each protein was normalized to that of 0 h in CHX and the results represent the mean ± SD (*n* = 3). *** *P*< 0.005 compared with control. **E.** Ishikawa cells were transfected with pcS4-3xHA-Ub and treated with E_2_ (10 nM) for 12 h followed by ALLN (25 μg/ml) for 12 h. Whole cell lysates (WCLs) were immunoprecipitated with anti-Ras antibody. **F.** AN3CA cells were transfected with pCMV-Flag-ER-α66, -ER-α66-deleted nuclear-localized signal (mut), or -ER-α36 expression vectors, and after 1 day, treated with E_2_ (10 nM) for 24 h. **G.** Ishikawa cells were transfected with control shRNA (shCon), ER-α36 shRNA (shERα-36 #1 or shERα-36 #3) and after 1 day, treated with E_2_ (10 nM) for 24 h. WCLs were analyzed by western blot analysis (A-C, E-G).

To investigate the effect of Ras stabilization by estrogen, we treated Ishikawa EC cells with E_2_. As shown by immunoblot analysis, endogenous pan-Ras protein was increased by E_2_ treatment in both dose-and time-dependent manners (Figure 1A and 1B). The regulation of Ras by E_2_ occurred at the level of protein stability rather than at the transcriptional level, as shown by the lack of significant changes in mRNA levels of K-, N-, and H-Ras (Supplementary Figure 1A). The half-life of pan-Ras, as determined by treatment with the protein synthesis inhibitor cycloheximide (CHX), was approximately 9 hours and was mostly blocked by E_2_ treatment (Figure 1C and 1D). The Ras proteins were subjected to the degradation by polyubiquitylation-dependent proteasomes [19, 20], but E_2_ reduced the level of Ras polyubiquitylation (Figure 1E).

Previous reports indicate that ER-α36 is expressed on the plasma membrane of EC cells [10, 12] and mediates membrane-initiated estrogen signaling. To determine whether ER-α36 involves the stabilization of Ras by estrogen, the AN3CA EC cell line, which expresses low levels of ER-α36 (Supplementary Figure 1B) and does not express ER-α66 [22], was transiently transfected with ER-α66, ER-α66-mut (an ER-α66 mutant with a deleted nuclear localization signal), or ER-α36 expression vectors. Levels of Ras were specifically increased by over-expressing ER-α36, but not ER-α66 or the ER-α66-mut, and were further increased by E_2_ treatment (Figure 1F). The role of ER-α36 in E_2_- stimulated Ras stabilization was confirmed by abolishment of the E_2_ effect on Ras stabilization by ER-α36 knockdown (Figure 1G). E_2_-BSA (bovine serum albumin), where E_2_ was covalently cross-linked to membrane-impermeable BSA, can only bind to the membraneous estrogen receptor [23], and cells treated with either E_2_ or E_2_-BSA similarly stabilized Ras at the same concentration (Supplementary Figure 1C).

The Ras levels were also increased by E_2_ treatment in MDA-MB-231 (ER-α66-negative breast cancer cells) and HEC1A (ER-α66-negative EC cells). However, the Ras levels were only weakly elevated by E_2_ in AN3CA cells, which express low levels of ER-α36 (Supplementary Figure 1D). Overall, these results showed that Ras was stabilized by E_2_ via membrane-associated ER-α36.

### PKCδ is specifically involved in K-Ras stabilization by estrogen

**Figure 2:**
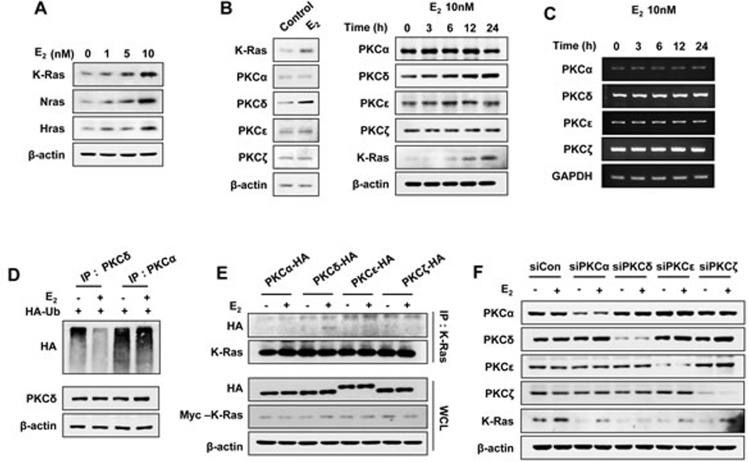
E_2_ induces K-Ras protein stabilization via PKCδ Ishikawa cells were treated with E_2_ in a dose- **A.** and time- **B.** dependent manner (left panel was treated E_2_ for 24 h). **B.** and **C.** PKC isotypes protein **B.** and mRNA **C.** expression levels were analyzed by western blot analysis and RT-PCR, respectively. **D.** Ishikawa cells were transfected with pcS4-3xHA-Ub and after 1 day, were treated with E_2_ (10 nM) for 12 h, followed by ALLN (25 μg/ml) for 12 h. WCLs were immunoprecipitated with anti-PKCδ or -PKCα antibody. **E.** Ishikawa cells were transfected with pcDNA3.1-Myc-K-Ras together with pHACE- PKCα, -PKCδ, -PKCε, or -PKCζ vector, and after 1 day, were treated with/without E_2_ (10 nM) for 30 min. WCLs were immunoprecipitated with anti-K-Ras antibody. **F.** Ishikawa cells were transfected with control, PKCα, PKCδ, PKCε, or PKCζ siRNA and after 1 day, treated with/without E_2_ (10 nM) for 24 h. WCLs were analyzed by western blot analysis (A-B, D-F).

The stabilization of Ras by estrogens occurred with different isotypes including K-, H-, and N-Ras (Figure 2A), which was similar to the finding that the stability of all of these Ras isotypes were increased via the Wnt/β-catenin signaling pathway [20]. We focused on K-Ras in the estrogen-induced Ras stabilization because K-Ras is associated with cellular transformation involving estrogens [24], and mutation or overexpression of K-Ras correlated with the development of EC [25]. As shown previously, estrogens activate the MAPK pathway via PKCδ in EC cells [12]; therefore, we tested the involvement of PKCδ in the stabilization of K-Ras by estrogen. Interestingly, we observed that protein expression of PKCδ, but not other PKC isotypes, was increased by E_2_ treatment of Ishikawa cells (Figure 2B; right panel shows time course data for E_2_ treatment). The increment of PKCδ expression by E_2_ was not caused by change of its mRNA level as shown by RT-PCR analyses (Figure 2C), but by protein stabilization by inhibiting its polyubiquitylation (Figure 2D).

In addition, PKCδ, but not PKCα, PKCε, or PKCζ, co-immunoprecipitated with K-Ras in an E_2_-dependent manner in EC (Figure 2E), which showed the specificity of the PKCδ binding to K-Ras. The role of PKCδ in K-Ras stabilization was further confirmed by specific knockdown of PKCδ, but not by knockdown of other PKC isotypes (Figure 2F).

The basal level of PKCδ expression in EC cell lines such as Ishikawa and HEC1A was higher than in the normal endometrial epithelial cell line EM-E6/E7/hTERT. Moreover, levels of PKCδ expression were proportional to the expression of pan-Ras and K-Ras as well as ER-α36 (Supplementary Figure 1B). By comparison, after specific knockdown of PKCδ in Ishikawa and HEC1A cells, which normally express high levels of both PKCδ and Ras, K-Ras expression was decreased compared to control (Supplementary Figure 2A). In addition, inhibition of *de novo* protein synthesis resulted in destabilization of K-Ras, which was significantly enhanced by PKCδ knockdown (t_1/2_=3 h *vs.*9 h) (Supplementary Figure 2B and 2C). In contrast with the knockdown effect, endogenous K-Ras levels were significantly increased by overexpression of PKCδ in RL95-2 and AN3CA cells, which normally express low levels of PKCδ (Supplementary Figure 2D).

We have shown that E_2_ induced K-Ras stabilization occurs through ER-α36 and PKCδ. To further elucidate the relationship between ER-α36 and PKCδ in K-Ras stabilization by estrogen, we examined whether K-Ras binds to ER-α36 or PKCδ. Results of immunoprecipitation experiments showed that PKCδ was detected with Myc-tagged K-Ras after E_2_ treatment and that association between K-Ras and PKCδ was significantly enhanced in cells where ER-α36 was overexpressed (Supplementary Figure 3A). In control cells, but not in shPKCδ cells, K-Ras levels were increased by overexpression of ER-α36 (Supplementary Figure 3B). However, the effect of PKCδ overexpression largely remained even after ER-α36 knockdown (Supplementary Figure 3C).

The abundance of endogenous K-Ras was reduced by knockdown of PKCδ and further knockdown of ER-α36 did not decrease K-Ras protein level (Supplementary Figure 3D). These results confirm that PKCδ functions downstream of ER-α36. Our results show that K-Ras protein is stabilized by estrogen via ER-α36 and that association occurred through enhancement of PKCδ binding affinity to K-Ras (Figure 7).

### PKCδ activity is essential for K-Ras stabilization by estrogen

**Figure 3:**
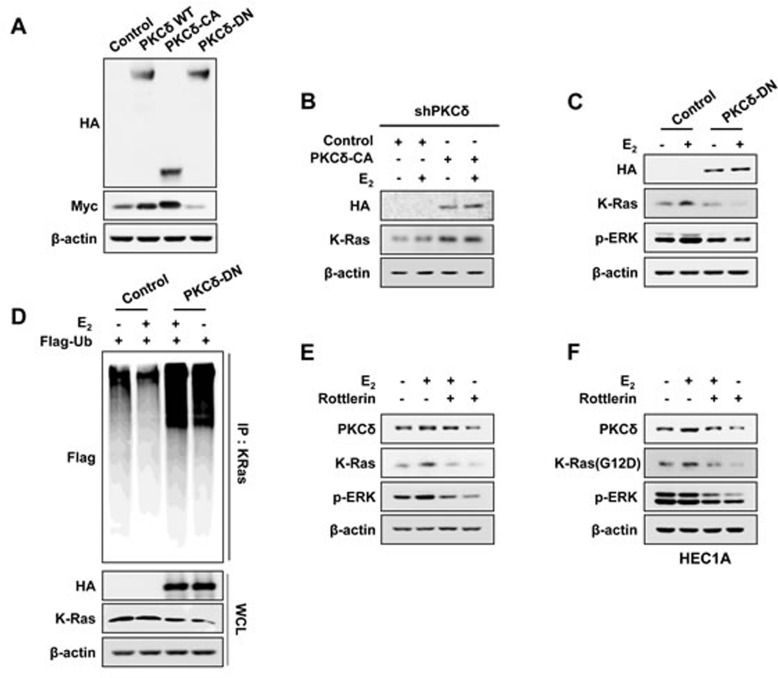
The effect of PKCδ activity on K-Ras stabilization by E_2_ **A.** Ishikawa cells were transfected with pcDNA3.1-Myc-K-Ras together with pHACE-PKCδ-WT, PKCδ-CA, or PKCδ-DN. **B.** The stable shPKCδ Ishikawa cells were transfected with pHACE or pHACE-PKCδ-CA and treated with E_2_ (10 nM) for 24 h after 1 day. **C.** Ishikawa cells were transfected with pHACE or pHACE-PKCδ-DN and after 1 day, were treated with E_2_ (10 nM) for 24 h. **D.** Ishikawa cells were co-transfected pcS4-3xflag-Ub and pHACE-PKCδ-DN, and after 1 day, the cells were treated with E_2_ (10 nM) for 12 h followed by ALLN (25μg/ml) for 12 h. WCLs were immunoprecipitated with anti-K-Ras antibody. Ishikawa cells **E.** and HEC1A cells **F.** were treated with E_2_ (10 nM) for 6 h followed by rottlerin (2 μM) for 18 h. WCLs were analyzed by western blot analysis (A-F).

Myc-tagged K-Ras was increased by overexpression of wild-type PKCδ, which was further increased and decreased by overexpression of catalytically active (PKCδ-CA) and dominant-negative PKCδ (PKCδ-DN), respectively (Figure 3A). Levels of endogenous K-Ras in shPKCδ-Ishikawa cells were significantly increased by overexpression of catalytically active PKCδ regardless of E_2_ treatment (Figure 3B).

In addition, E_2_-induced K-Ras stabilization was abolished by overexpression of dominant-negative PKCδ, and the activation status of ERK was proportional to the levels of K-Ras (Figure 3C).

We confirmed that the mechanism of K-Ras stabilization by E_2_ requiring PKCδ activity was through inhibition of polyubiquitylation-dependent proteasomal degradation by showing increment of K-Ras polyubiquitylation by PKCδ-DN overexpression, independent of E_2_ treatment (Figure 3D). Finally, the role of PKCδ activity in K-Ras stabilization was confirmed by abolishment of E_2_-induced K-Ras stabilization by the PKCδ specific inhibitor, Rottlerin (Figure 3E). We also found that the stability of the oncogenic mutant K-Ras (G12D) was increased by estrogens via PKCδ as shown by rottlerin treatment (Figure 3F). In summary, PKCδ activity is involved in the increment of K-Ras stability by estrogen signaling.

### PKCδ-stimulated K-Ras stabilization by estrogen is involved in transformation of EC cells

**Figure 4:**
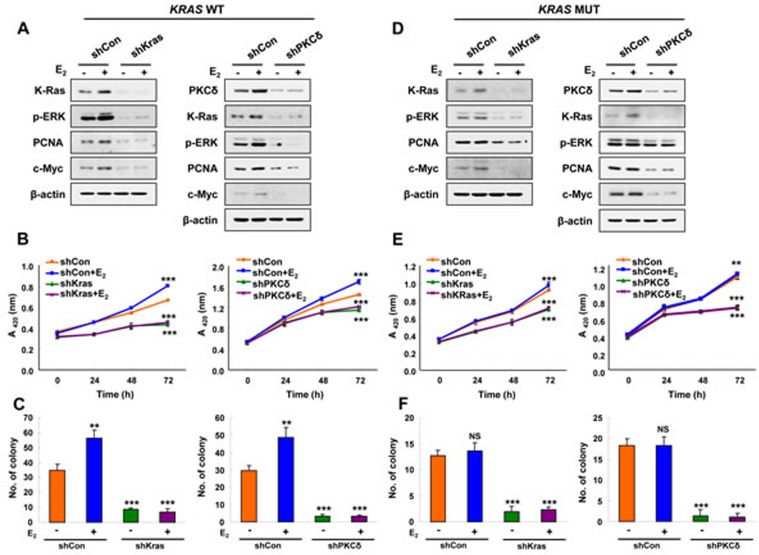
The effects of PKCδ or K-Ras knockdown on E_2_-induced Ras-ERK pathway activation and cellular transformation **A.**, **B.**, and **C.** Stable shCon, shK-Ras, or shPKCδ Ishikawa cells. **D.**, **E.**, and **F.** Stable shCon, shK-Ras, or shPKCδ HEC1A cells. (A and D) Cells were treated with/without E_2_ (10 nM) for 24 h. WCLs were analyzed by western blot analysis. (B and E) MTT assays were performed every 24 h for 72 h after E_2_ (10 nM) treatment. The results represent the mean ± SD (*n* = 3). **P < 0.01 and ***P < 0.005. (C and F) Cells were treated with E_2_ (10 nM) every 3 days for 14 days. Colony numbers were counted. The results represent the mean ± SD (*n* = 3). **P < 0.01 and ***P < 0.005 compared with control. NS = not significant compared with control.

To understand role of K-Ras stabilization via PKCδ in the transformation of EC cells, we generated cells with stable knockdown of K-Ras or PKCδ by infection with shK-Ras and shPKCδ lentiviruses, respectively. ERK activity and expression of PCNA and c-Myc were increased along with K-Ras stabilization by E_2_ in Ishikawa cells, and these E_2_ effects were abolished in shK-Ras cells (Figure 4A, left). Similar results were observed in shPKCδ cells (Figure 4A, right). Cell proliferation was increased by 43% at 72 hours after E_2_ treatment, and the E_2_-induced cell proliferation was totally abolished by knockdown of K-Ras or PKCδ and (Figure 4B). Similarly, numbers of transforming foci were significantly increased by E_2_ treatment, and basal as well as the E_2_-induced foci formation were abolished in the cells with knockdown of K-Ras or PKCδ (Figure 4C and Supplementary Figure 4A).

The oncogenic K-Ras mutant was also stabilized by E_2_ treatment, but this was abolished, together with reduction of ERK activity, by knockdown of the mutant K-Ras or PKCδ (Figure 4D). Cell proliferation and transforming focus formation were not increased by E_2_ in HEC1A cells harboring mutant *K*-*Ras*; however, those were significantly reduced by K-Ras or PKCδ knockdown (Figure 4E, 4F and Supplementary Figure 4B). Consequently, our results showed that PKCδ is important for proliferation and transformation induced by K-Ras stabilization.

### PKCδ inhibitor suppresses E_2_-induced K-Ras stabilization and tumor growth in a mouse xenograft model

**Figure 5:**
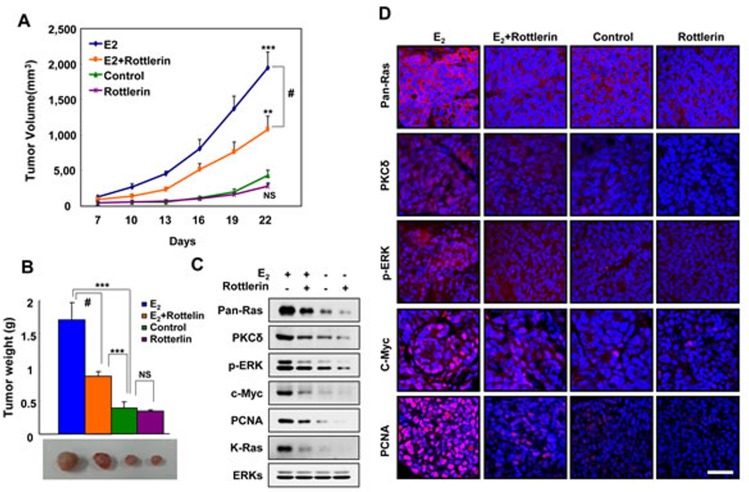
Effects of a PKCδ inhibitor on E_2_-induced tumor growth, and PKCδ and K-Ras stabilization in the mouse xenograft model **A.** Ishikawa cells were subcutaneously injected along with a 1.7 mg/60-day E_2_ pellet into athymic female nude mice. Two days later, rottlerin was administered intraperitoneally at 5 mg/kg once daily. Tumor size was measured with Vernier calipers every 3 days. **B.** The mice were sacrificed after 22 days, and tumor weights were measured by an electronic balance. The results represent the mean ± SD (*n* = 8). **P < 0.01, ***P < 0.005, NS = not significant, and #, *P*< 0.01 E_2_ vs E_2_+rottlerin. The results represent the mean ± SD. Gross images from representative tumors are shown at the bottom. **C.** WCLs were analyzed by western blot analysis. **D.** Paraformaldehyde-fixed paraffin-embedded sections were subjected to immunofluorescence analyses and images were captured by laser scanning confocal microscopy. Scale bar, 40 μm.

To identify the role of K-Ras stabilization involving PKCδ in EC tumorigenesis *in vivo*, we examined whether inhibition of PKCδ blocked estrogen-induced tumor growth of EC cells in a mouse xenograft model. We used Ishikawa cells harboring wild-type K-Ras to monitor the unique effect of stabilization, and not the effect of oncogenic activation of K-Ras, by estrogens on tumor growth. Tumor growth was monitored either by volume or weight, and was significantly increased in the xenografted mice injected with cells treated with the E_2_ pellet. The volumes and weights of tumors for E_2_-induced xenograft were significantly reduced by co-treatment with the PKCδ inhibitor rottlerin. Furthermore, tumor volume and weight were reduced by 55% and 50%, respectively, in mice co-treated with rottlerin (Figure 5A and 5B).

To verify whether the mechanism for E_2_-induced Ras stabilization involving PKCδ was involved in tumorigenesis *in vivo*, we monitored levels of PKCδ, pan-Ras, p-ERK, and c-Myc in the tumors by both western blot and immunofluorescence analyses. The levels of pan-Ras, PKCδ, and p-ERK were increased by E_2_ but blocked by rottlerin (Figure 5C and 5D). The levels of PCNA correlated with pan-Ras and PKCδ by western blot and immunofluorescence analyses. Therefore, we found that PKCδ stimulates EC tumorigenesis by E_2_-induced K-Ras stabilization *in vivo*.

### The expression of Ras and PKCδ was up-regulated in human endometrial carcinoma

**Figure 6:**
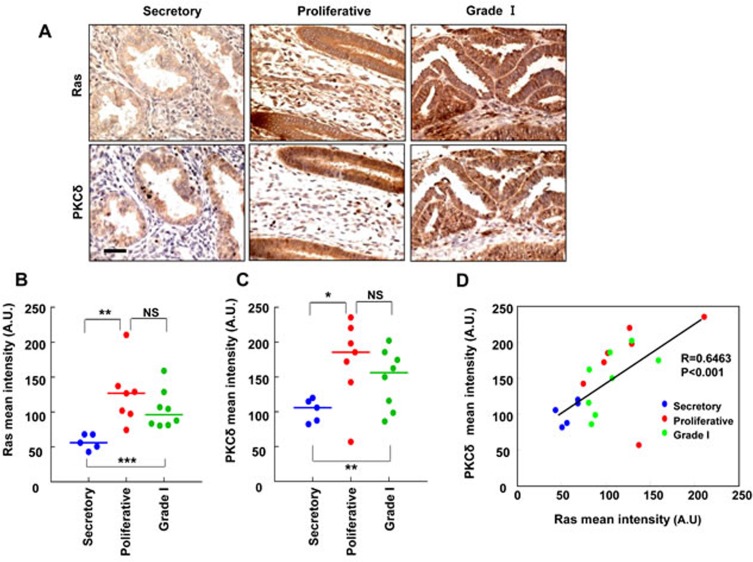
The relative quantification of Ras and PKCδ protein levels in human endometrial tissues **A.** Ras or PKCδ in TMA serial sections of normal endometrium (secretory and proliferative stages) and endometrial carcinoma (Grade I) were evaluated by immunohistochemical analyses and microscopy. Scale bar, 40 μm. **B.** and **C.** Quantitative analyses of immunohistochemical staining were performed using HistoQuest software. The results represent the mean ± SD. *P < 0.05, **P < 0.01 and ***P < 0.005, and NS = not significant. **D.** The correlation between Ras and PKCδ protein levels was analyzed by Pearson's correlation analysis with a coefficient of 0.6463 (*p* < 0.001).

To investigate the involvement of Ras and PKCδ in human EC, we used tissue microarrays from 12 cases of normal tissue (5 secretory stage and 7 proliferative, respectively) and 8 cases of grade I tumors. The normal tissue arrays included samples from both secretory stage and proliferative endometrium to check variation due to the menstrual cycle. Immunohistochemistry for pan-Ras and PKCδ was performed, and representative images of the staining are presented (Figure 6A). Both Ras and PKCδ expression was more abundant in the grade I endometrial carcinoma and proliferative phase of normal endometrium compared with the secretory phase of normal endometrium (Figure 6B and 6C). The intensities of Ras and PKCδ staining showed similar patterns in the epithelium of the endometrial glands, and positive correlation was shown for Ras and PKCδ in between proliferative phase of normal endometrium and grade I endometrial carcinoma (Figure 6D).

## DISCUSSION

Ras proteins are important for various cellular functions including cell proliferation, differentiation, and survival. The regulation of Ras proteins and their activities are controlled via switching of their GDP and GTP binding forms by extra- and intra-cellular signaling. Activated GTP-bound Ras proteins increase proliferation signaling through downstream pathways including the Raf-ERK and PI3 kinase-Akt pathways [26]. Aberrant activation of Ras proteins by mutations that fix Ras as a GTP binding form promotes various human tumors. *K-Ras* mutations are most common among the Ras isotypes and are found in various human cancers at frequencies as high as 40-50% in colorectal cancer and 90% in pancreatic cancer [27, 28]. The *K*-*Ras* mutation is found at a much lower frequency of about 15% in human EC [29].

Ras activity is mainly controlled by the GDP-GTP switch, but it also controlled by membrane localization [30]. Recently, the stabilization of Ras protein has been shown as an alternative mechanism for Ras activity control. Ras protein stability increased by the Wnt/β-catenin signaling has been shown to be directly involved in colorectal tumorigenesis and cancer stem cell activation [19-21]. In addition, up-regulation of Ras has been observed in several cancers including colorectal and breast [31, 32], and its increased expression has been associated with neoplastic transformation.

**Figure 7:**
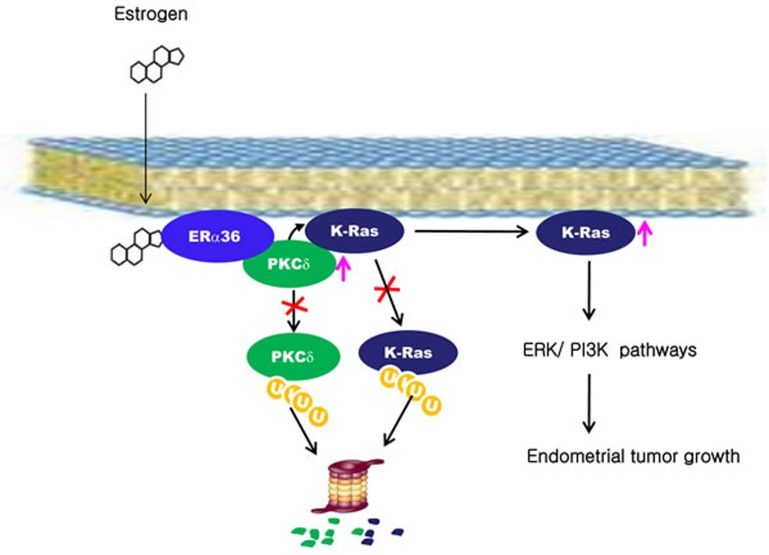
A proposed model for K-Ras stabilization by estrogen in endometrial tumorigenesis Estrogen stabilizes PKCδ by inhibiting ubiquitylation-dependent proteasomal degradation. PKCδ binds to K-Ras via ER-α36 in a manner dependent upon estrogen and subsequently inhibits K-Ras degradation. The stabilized K-Ras stimulates endometrial tumor growth via the ERK and PI3 kinase signaling pathways.

In this study, we also found that Ras protein levels were high in the proliferative stage of normal endometrium as well as in EC tissues. The Ras isotypes including K-Ras, N-Ras, and H-Ras, were all subjected to stability increments by estrogens via the mechanism involving membraneous ER-α36 followed by activation of PKCδ (Figure 7).

PKCδ was specifically involved in the K-Ras stabilization by estrogen and the estrogen-induced K-Ras stabilization was abolished by knockdown of PKCδ, but not by knockdown of other PKC isotypes. The significance of PKCδ in K-Ras stabilization by estrogens was indicated by complete abolishment of the estrogen effect by PKCδ knockdown. Tumors with wild-type Ras and aberrant activation of the PI3K or ERK pathways have been shown to require PKCδ activity for proliferation or survival [33], indicating the important role of PKCδ activity in cellular transformation. We identified that PKCδ activity is important in the K-Ras stabilization by estrogens; the stabilization increased through ER-α36 (Figure 7). The ubiquitination assays for both PKCδ and K-Ras showed that both of the two proteins were increased via inhibition of polyubiquitylation-dependent proteasomal degradation. Furthermore, PKCδ activity was shown to be required for the inhibition of ubiquitination and subsequent stabilization of Gadd45, which correlated with the role of PKCδ in K-Ras stabilization [34].

The stabilization of K-Ras by estrogens via PKCδ was correlated with the proliferation and transformation of EC cells as shown by K-Ras and PKCδ knockdown, respectively. The suppression of the estrogen induced tumor growth of EC cells by the PKCδ inhibitor rottlerin implicated K-Ras stabilization by estrogens in the growth of EC. Inhibition of the xenograft tumor growth by rottlerin provides *in vivo* evidence for the role of PKCδ in the tumor growth. The role of K-Ras stabilization via PKCδ was confirmed *in vivo* by both immunoblot and immunohistochemical analyses of human EC tissues. Our results suggest that PKCδ is a potential therapeutic approach for development of anti-cancer drugs for treatment of ECs.

## MATERIALS AND METHODS

### Cell lines, culture and reagents

HEC1A, AN3CA, and KLE cell lines were provided by Dr. Churl Ki Min (Ajou University, Suwon, South Korea), and the RL95-2 cell line was provided by Dr. Jong-Min Kim (Dong-A University, Pusan, Korea). The Ishikawa cell line was a gift from Dr. Hyung Sik Kim (Pusan National University, Pusan, Korea). The EM-E6/E7/hTERT cell line was originated by Mizumoto and colleagues [35] and obtained from Dr. Paul J. Goodfellow (Ohio State University, Columbus, OH). ECC-1 cells were purchased from the ATCC. All cell lines were maintained in Dulbecco's Modified Eagle Medium: Nutrient Mixture F-12 (DMEM /F12; Gibco) supplemented with 10% fetal bovine serum (FBS) (Gibco), 1% penicillin-streptomycin (Gibco) at 37°C in a humidified atmosphere of 5% CO_2_. For E_2_ treatment, cells were maintained in phenol red-free DMEM/F12 (Gibco) with 5% charcoal-stripped FBS for 1 day followed by serum-free medium for 24 h before the experiment. The E_2_ and E_2_-BSA (bovine serum albumin) were purchased from Sigma. E_2_-BSA was prepared according to the protocol for removing free E_2_ as previously described [36]. Cycloheximide (CHX) and *N*-acetyl-leucinyl-leucinyl-norleucinal (ALLN) were purchased from Sigma.

### Establishment of stable cell lines

shRNA PKCδ for the stable knockdown of human PKCδ expression [37] was obtained from Mary E. Reyland (University of Colorado, Denver, CO). Stable knockdown of K-Ras was accomplished using lentiviral constructs containing short hairpin RNA (shRNA) for human *K-Ras* (Sigma). The lentiviral vectors were transfected into HEK-293T cells with the packaging plasmids psPAX2 and pMD2.G (a gift from Dr. KunLiang Guan, University of California, San Diego, CA) [38] and using Lipofectamine reagent (Invitrogen). The virus particles were harvested at 24 h after transfection. Then, fresh media was added to the cells, which were harvested at 48 h. EC cell lines were infected with lentivirus-containing media with polybrene (8 μg/ml) for 6 h, followed by replacement with fresh media. Cell lines were selected in media with puromycin (1 μg/ml). Stable knockdown of human PKCδ was carried out using lentiviral constructs.

### Quantitative real-time PCR

Total RNA was extracted from cells using TRIzol (Invitrogen). Transcripts were measured by quantitative real-time PCR (qRT-PCR) analysis using the Qiagen StepOnePlus qRT-PCR system according to the manufacturer's instructions (Qiagen). All RT-PCR analyses were performed using five independent RNA sets. The relative expression of each transcript was normalized to *β-actin*. The following primer sets were used: *K-Ras*, forward 5′-GTATAGAAGGCATCATCAACAC and reverse 5′-AAACAGGCTCAGGACTTAG; *H-Ras,* forward 5′-AGACTTGGTGTTGTTGATGG and reverse 5′-GGAAGCAGGTGGTCATTG-3′; *N-Ras* forward 5′-CCATCATCACTGCTGTTGA-3′ and reverse 5′-AAGAGTTACGGGATTCCATTC-3′; *β-actin*, forward5′-ATAGCACAGCCTGGATAGCAAC-3′ and reverse 5′-AATCTGGCACCACACCTTCTAC-3′.

### Plasmids and transfection

The ER-α36-specific shRNA expression vector 231/sh36 (3-1) (#1), 231/sh36 (1-7) (#3), control vector expressing shRNA for luciferase 231/shluc, and pCB6-HA-ER-α36 were provided by Dr. Zhaoyi Wang (Creighton University Medical School, Omaha, NE) [39]. pCMV-Flag-ER-α36 was generated by PCR of pCB6-HA-ER-α36 using primer sets (forward 5′-GAATTCAATGGCTATGGAATCTGCC-3′ and reverse 5′-GAATTCTTAGACACGAGGAAACCA-3′) followed by cloning into pCMV. pCMV-Flag-ER-α66 [40] was provided by Dr. W. Lee Kraus (Cornell University, Ithaca, NY). The sequence coding the nuclear localizing signal (NLS, 250-303 amino acids of ER-α66) was deleted with PCR-based sited-directed mutagenesis of the pCMV-Flag-ER-α66. pHACE plasmids coding for HA-tagged PKCδ-WT, PKCδ-DN, PKCδ-CA, PKCα, PKCε, and PKCζ [41] were provided by Dr. Jang-Soo Chun (Gwangju Institute of Science and Technology, Kwangju, Korea). EC cells were seeded at 60 mm culture dishes (1×10^5^ cells) and culture for 24 h before transfection. Transfection of plasmids was performed with Lipofectamine according to the manufacturer's instructions.

### *In vivo* ubiquitylation assay and immunoblot analysis

*In vivo* ubiquitylation assays and immunoblot analyses were performed as previously described [19]. Briefly, N-ethylmaleimide (10 mM, Sigma) was added to the radioimmunoprecipitation assay (RIPA) buffer (Upstate Biotechnology). The lysates were incubated with the indicated antibodies and Protein G agarose at 4°C for 12 h, and the beads were washed three times with cold RIPA buffer. Immunoblot analysis was performed as previously described [19]. Primary antibodies were obtained from the following sources; anti-pan-Ras (Millipore); -K-Ras, -N-Ras, -H-Ras, -β-actin, -p-ERK, -p-AKT, -PKCδ, -PKCα,-PCNA, and -c-Myc (Santa Cruz Biotechnology); -HA, -PKCε, and -PKCζ (Cell Signaling Technology); -Flag (Sigma); and -ER-α36 (Cell Applications). Secondary antibodies were horseradish peroxidase (HRP)-conjugated anti-mouse (Cell Signaling Technology) and HRP-conjugated anti-rabbit (Bio-Rad).

### MTT and focus formation assays

For the MTT assay, cells were plated at 24-well plate (1.7×10^4^ cells) and treated with/without E_2_ for 72 h. MTT reagent (3-(4,5-dimethylthiazol-2-yl)-2,5-diphenyltetrazolium bromide; AMRESCO) was diluted in phenol red-free DMEM/F12 at a concentration of 0.25 mg/ml. Cells were incubated for 2 h at 37°C. Medium was removed and insoluble formazan was solubilized with 500 μl dimethyl sulfoxide (DMSO; Sigma) for 30 min. Formazan product absorbance was determined at 420 nm.

For focus formation assay, cells were seeded at 12-well plate (500 cells). The cells were treated with E_2_ in phenol red-free DMEM/F12 for 14 days, and in case of Ishikawa cells, 0.5% charcoal-stripped FBS was added. Media were changed at every 3 days. After 14 days, cells were stained with 0.5% crystal violet in 20% ethanol.

### *In vivo* tumor xenograft assay

Approximately 5-week-old female *Balb Cnu/nu* mice were purchased from Orientbio Inc. (Seongnam, Korea). Mice were maintained as previously described [42]. An E_2_ pellet (1.7 mg/60-day release; Innovative Research of America) was implanted subcutaneously before the injection of Ishikawa cells. Five days later, Ishikawa cells (1.5×10^7^) in 100 μL of DMEM/F12:Matrigel (1:1) were subcutaneously injected into the dorsal flank of each mouse. Two days later, 5 mg/kg of rottlerin in DMSO was administered intraperitoneally to the mice daily for 22 days, and the control mice were given DMSO in the same manner. Tumor volumes and body weights of mice were measured at every 3 days. Tumors were measured with Vernier calipers, and tumor volumes were calculated by the formula π/6×length×width×height. The mice were sacrificed under anesthesia, and the tumors were collected for further analysis.

### Immunohistochemical analysis

Tumor samples were fixed in 4% paraformaldehyde-fixed and embedded in paraffin wax according to standard procedures. Tissue sections (4 μm each) were deparaffinized in xylene and dehydrated through a gradient concentration of alcohol. For antigen retrieval, the slides were autoclaved in buffer (10 mM sodium citrate pH 6.0, Sigma-Aldrich). Sections were preincubated in phosphate-buffered saline (PBS) and then blocked with 5% BSA in PBS for 30 min at room temperature. The sections were incubated with the following primary antibodies overnight at 4°C: anti-pan-Ras (1:100, Millipore), -PKCδ (1:100, Santa Cruz Biotechnology), -PCNA (1:1000, Santa Cruz Biotechnology)-c-MYC (1:100, Santa Cruz Biotechnology) -p-ERK (1:20, Cell Signaling Technology, -p-Akt (1:20, Cell Signaling Technology).

For immunofluorescence staining, the sections were then incubated with Alexa Fluor 488- or Alexa Fluor 555-conjugated secondary antibodies (Invitrogen) at room temperature for 1 h followed by counterstaining with 4′,6-diamidino-2-phenylindole (DAPI) (Boehringer Mannheim) and mounted in Gel/Mount medium (Biomeda Corporation). Visualization of the fluorescence signal was performed by confocal microscopy (Carl Zeiss LSM700, Oberkochen, Germany).

Tissue Microarrays (TMAs) for normal and cancerous endometrial tissues were purchased from US Biomax. For immunoperoxidase staining, endogenous peroxidase was blocked with 0.345% H_2_O_2_ (Samchun Chemicals, Pyeongtaek, Korea) for 15 min, followed by incubation with biotin-conjugated secondary antibodies for 1 h at room temperature, washing and incubation with an Avidin-Biotin complex (Vector Laboratories) for 30 min. The brown color indicative of peroxidase activity was developed by 3, 3-incubating with diaminobenzidine (DAB) staining solution (Vector Laboratories) followed by counter staining with Mayer's hematoxylin (Muto Chemicals, Tokyo, Japan). The DAB-stained preparations were visualized with a Nikon bright-field microscope (Nikon TE-2000U, Tokyo, Japan). Relative staining intensities were quantified using HistoQuest software (TissueGnostics, Vienna, Austria).

### Statistical analysis

Data are presented as mean ± standard deviation. Student's t-test was performed using Microsoft Excel spreadsheets and GraphPad Prism. Statistical significance is indicated in the figures as follows: *P < 0.05, ** *P* < 0.01, and ***P < 0.005.

## SUPPLEMENTARY MATERIAL FIGURES AND TABLE


