# Supplementary material for: K-Ras stabilization by estrogen via PKCδ is involved in endometrial tumorigenesis
Source: Oncotarget. 2015 May 8;6(25):21328–40. doi: 10.18632/oncotarget.4049 (PMC4673268; doi:10.18632/oncotarget.4049)
Supplement: Supplementary file 1 [file oncotarget-06-21328-s001.pdf]

K-Ras stabilization by estrogen via PKCδ is involved in endometrial tumorigenesis

SUPPLEMENTAL MATERIAL

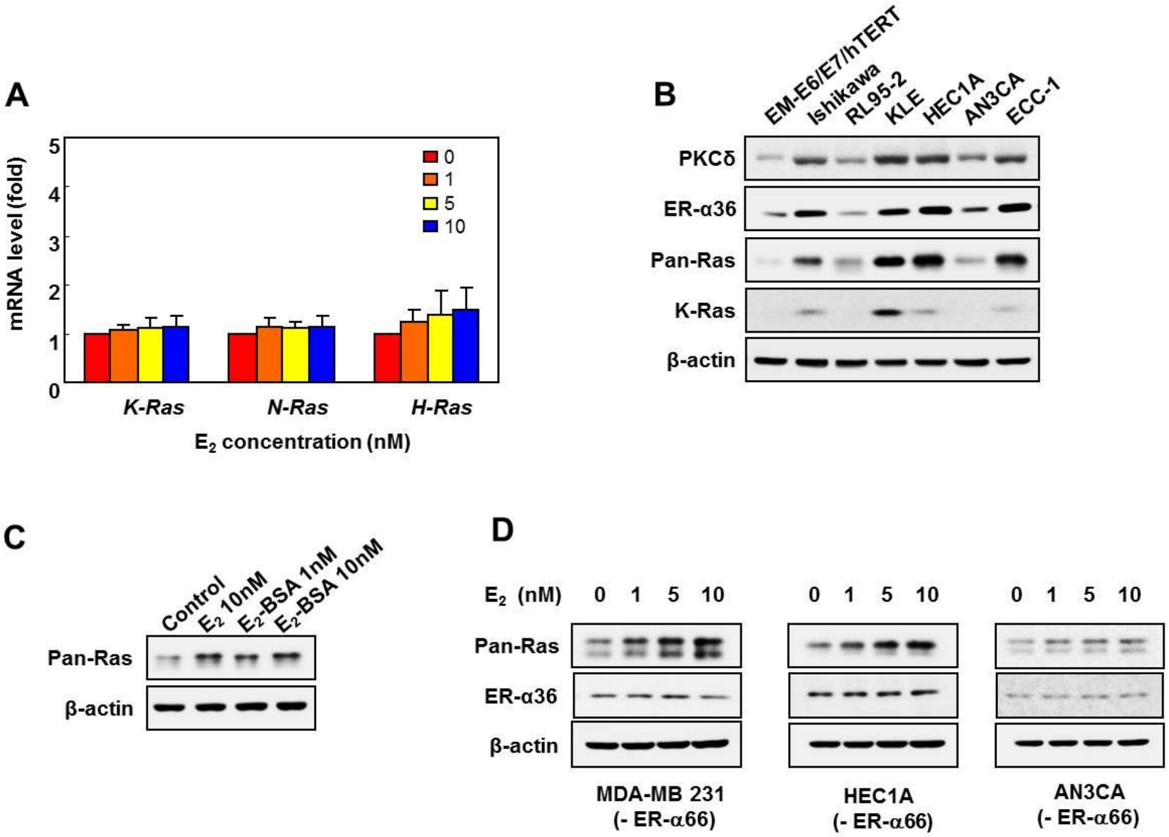

Supplementary Figure 1 : Ras protein is stabilized, but not mRNA, by E_2 via ER-α36. (A) Quantitative PCR analysis of the mRNA expression of *K-Ras*, *N-Ras*, and *H-Ras* in Ishikawa cells treated with E_2 in a dose-dependent manner for 24 h. The results represent the mean ± SD shown for five independent experiments. (B) WCLs of normal endometrial and EC cell lines were analyzed by western blot analysis. (C) Ishikawa cells were treated with E_2 or BSA-E_2 for 24 h. (D) MDA-MB231, HEC1A, and AN3CA cells were treated E_2 in a dose-dependent manner for 24 h. WCLs were analyzed by western blot analysis (B-D).

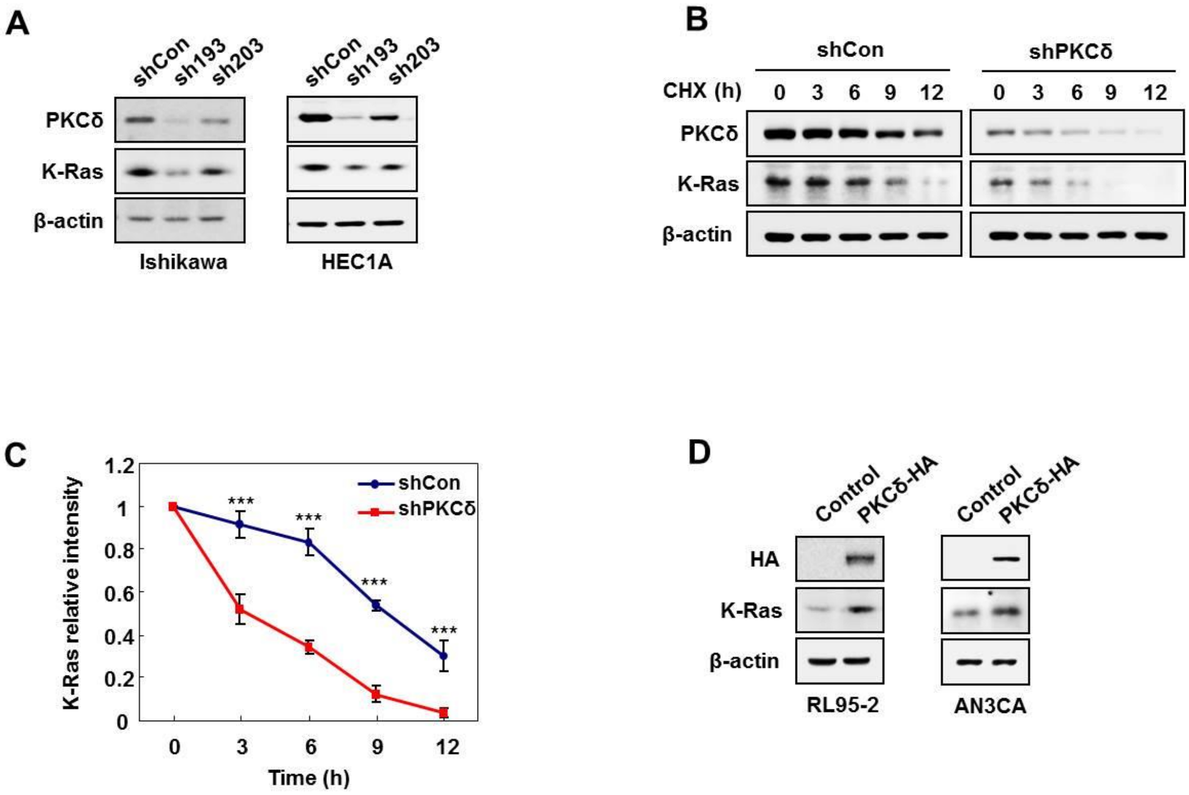

Supplementary Figure 2 : Effect of PKCδ on K-Ras protein level. (A) Ishikawa and HEC1A cell lines were infected PKCδ knockdown virus and established stable cell lines. (B) Stable shCon or shPKCδ Ishikawa cells were treated with CHX (50 μg/ml) for the indicated time periods. (C) The graph shows the quantification of the K-Ras band intensities of the blot (B) relative to β-actin control. Band intensity of each protein was normalized to that of 0 h in shCon or shPKCδ, and the results represent the mean ± SD (n = 3). *** $P< 0.005$ compared with shCon. (D) RL95-2 and AN3CA cells were transfected with pHACE-PKCδ. WCLs were analyzed by western blot analysis (A, B, D).

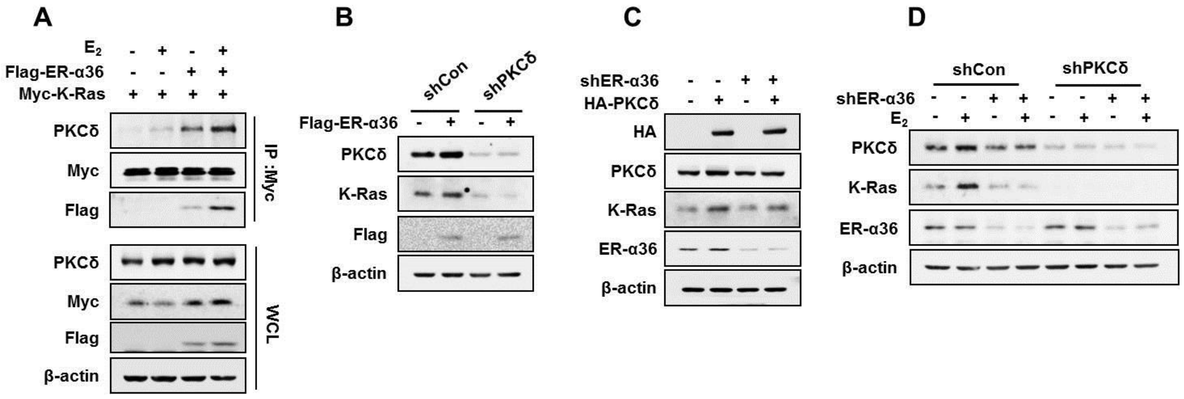

Supplementary Figure 3 : PKCδ is downstream of ER-α36 on E_2-mediated K-Ras stabilization. (A) Ishikawa cells were transfected with pcDNA3.1-Myc-K-Ras together with pCMV-Flag-ER-α36, and after 1 day, were treated with/without E_2 (10 nM) for 30 min. WCLs were immunoprecipitated with anti-Myc antibody. (B) The stable shPKCδ Ishikawa cells were transfected with pCMV or pCMV-Flag-ER-α36. (C) Ishikawa cells were transfected with the pHACE-PKCδ WT together with shER-α36 (#3). (D) The stable shCon or shPKCδ Ishikawa cells were transfected with the control shRNA (shCon) or shER-α36 (#3) and after 1 day, the cells were treated with/without E_2 (10 nM) for 24 h. WCLs were analyzed by western blot analysis (A-D).

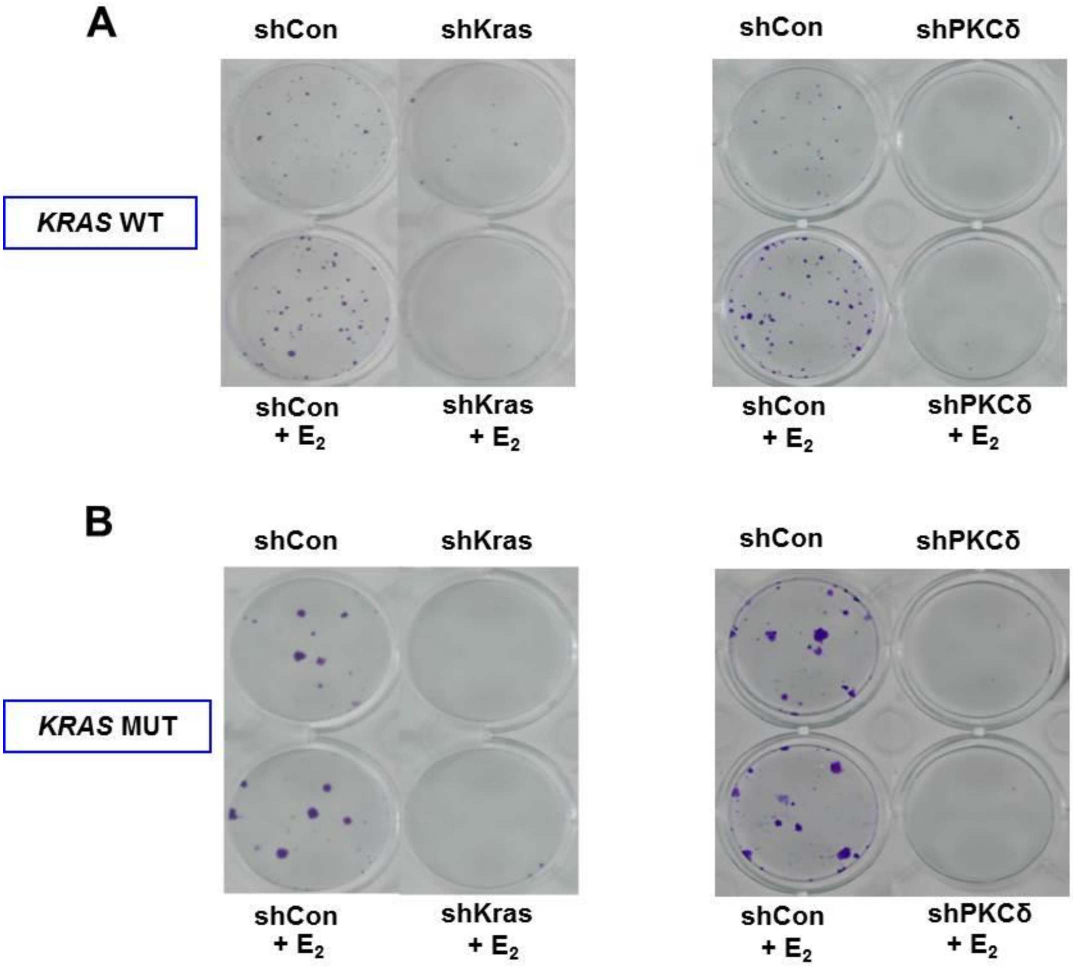

Supplementary Figure 4 : Stable shCon, shK-Ras and shPKCδ (A) Ishikawa and (B) HEC1A cell lines were grown with/without E_2 (10 nM) for 14 days. Pictures of crystal violet-stained transformed colonies are shown.

Supplementary Table 1: Primers and siRNA sequences for PKC isotypes

gene	primers
PKCα	5′-CGGGCTTTCAGATCCTTATGT-3′
	5′-CAGAGGGACTGATGACTTTGTT-3′
PKCδ	5′-ACCATAAATGCCGGGAGAAG-3′
	5′-GATGCCCTTGCTGTGTAGAA-3′
PKCε	5′-CCGTACCTTACCCAACTCTACT-3′
	5′-CCTCCTGGTTGATCTGCTTTAC-3′
PKCζ	5′-GGTGCATGATGACGAGGATATT-3′
	5′-CGTAGTCTGTGAGCTTGATGTG-3′

gene	target sequence
PKCα (NM002737)	5′-GUCACAGUACGAGAUGCAA-3′
	5′-CAACGUACCCAUUCCGGAA-3′
PKCδ (NM212539)	5′-CCAUGUAUCCUGAGUGGAA-3′
	5′-AAAGAACGCUUCAACAUCG-3′
PKCε (NM005400)	5′-AAGAUCAAAAUCUGCGAGGCC-3′
	5′-AAGAUCGAGCUGGCUGUCUUU-3′
PKCζ (NM002744)	5′-GGAGACAGAUGGAAUUGCUUACAUU-3′
	5′-GUUCGAAGGCUUUGAGUAUAU-3′
